# A case report of spontaneous umbilical enterocutaneous fistula resulting from an incarcerated Richter’s hernia, with a brief literature review

**DOI:** 10.1186/s12893-017-0216-z

**Published:** 2017-02-13

**Authors:** Wei Chen, Lei Liu, Hui Huang, Mianxu Jiang, Tao Zhang

**Affiliations:** 1 Key Laboratory for Molecular Diagnosis of Hubei Province, The Central Hospital of the Wuhan, Tongji Medical College, Huazhong University of Science and Technology, Wuhan, 430014 People’s Republic of China; 2 Department of Gastrointestinal Surgery, The Central Hospital of the Wuhan, Tongji Medical College, Huazhong University of Science and Technology, Wuhan, 430014 People’s Republic of China

**Keywords:** Enterocutaneous fistula, Umbilical, Fistulography, Richter’s hernia

## Abstract

**Background:**

Richter’s hernia is a high-risk ischaemic gastrointestinal disorder that is typically diagnosed in a delayed manner due to a lack of obvious symptoms. Spontaneous umbilical enterocutaneous fistula (ECF) resulting from an incarcerated Richter’s hernia is extremely rare.

**Case presentation:**

A 62-year-old female presented with a chief complaint of recurrent umbilical region infection for the preceding 20 months with no symptoms of ileus. Preoperative CT and fistulography revealed an incarcerated Richter’s hernia complicated by an ECF. Exploratory laparotomy revealed a loop of the distal ileum adherent to the umbilical region that was retrieved back into the abdominal cavity. Side-to-side ileo-ileal anastomosis was performed using a 75 mm linear stapler to remove the affected ileum segment. The internal hernia ring was closed using plication sutures instead of via mesh repair due to the patient’s small defect and infection risk.

**Conclusion:**

Richter’s hernia can be observed at any age but is particularly common in frail, elderly patients. This nonspecific clinical and laboratory findings of this condition are associated with a high misdiagnosis rate, resulting inrelatively high mortality. Abdominal CT and gastrointestinal imaging are recommended if Richter’s hernia is suspected. Timely surgical intervention is crucial for reducing mortality and improving prognosis.

## Background

Richter’s hernia has been well established as a specific type of enterocele since the firstconcrete definition of this condition was provided by a German surgeon,August Gottlied Richter, in 1785 [1]. This condition is associated with a high risk of ischaemic gastrointestinal disorders in which the advanced symptoms of ileus and/or perforation are directly related in most cases to the proportion of the circumference of the bowel wall that is entrapped [2]. Only when approximately two-thirds of the circumference of the intestinal wall is involved do symptoms of bowel obstruction gradually become apparent. Therefore, patients with Richter’s hernia are highly unlikely to seek treatment in a timely manner; this delay can allow bowel necrosis to develop, with the secondary formation of an enterocutaneous fistula (ECF). To date, Richter’s hernia with strangulation has more commonly been reported in the femoral ring and at trocar sites after laparoscopic procedures [3]. However, few literature reports have discussed ECF following an incarcerated spontaneous abdominal wall hernia [4, 5]. Here, we describe the history of a 62-year-old patient with an umbilical region hernia and the formation of an ECF 20 months after incarceration and discuss a systematic review of the literature.

## Case presentation

A 62-year-old female presented to the outpatient department of Wuhan Central Hospital of Tongji Medical College in September 2015with a complaint of recurrent infections in the umbilical region. She reported abdominal pain similar to a burning sensation that accompanied the discharge of faecal matter. These symptoms and signs waxed and waned but lasted for 5years. Our attention was piqued by the fact that the patient’s family described the patient as an individual who cried easily. The patient had no history of diarrhoea, constipation or other abdominal disturbances. No surgical treatment was mentioned in her prior medical history.

**Fig. 1:**
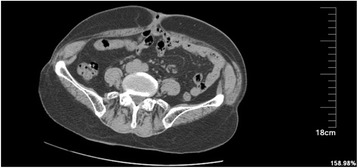
A CT scan showing a loop of intestine lumen was entrapped in the anterior abdominal wall

**Fig. 2:**
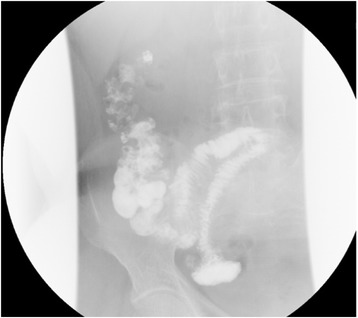
The digital radiography showed that the distal ileum approximately 40cm from the ileocaecal junction was entrapped

Acoordinated physical examination revealed normal vital signs. An external fistula was located in the umbilical region with redness of the surrounding skin. Morphological examination indicated that fistula secretions mainly consisted of small intestinal juice. The abdominal wall was soft, with no tenderness. Bowel sounds were regular. *Escherichia coli* and *Enterococcus faecalis* were detected in the fistula secretion culture. Other findings from laboratory examinations were normal. A CT scan of the abdomen revealed that part of the intestinal wall was adhered to the abdominal wall in the navel region, although no bowel obstruction was detected (Fig. 1). A presumptive diagnosis of ECF was reached; this diagnosis was mainly based on digital radiography of the fistulous tract conducted using iopamidol-370 as a contrast agent. This procedure was performed under local anaesthesia and revealed that the distal ileum approximately 40 cm from the ileocaecal junction was entrapped (Fig. 2).

**Fig. 3:**
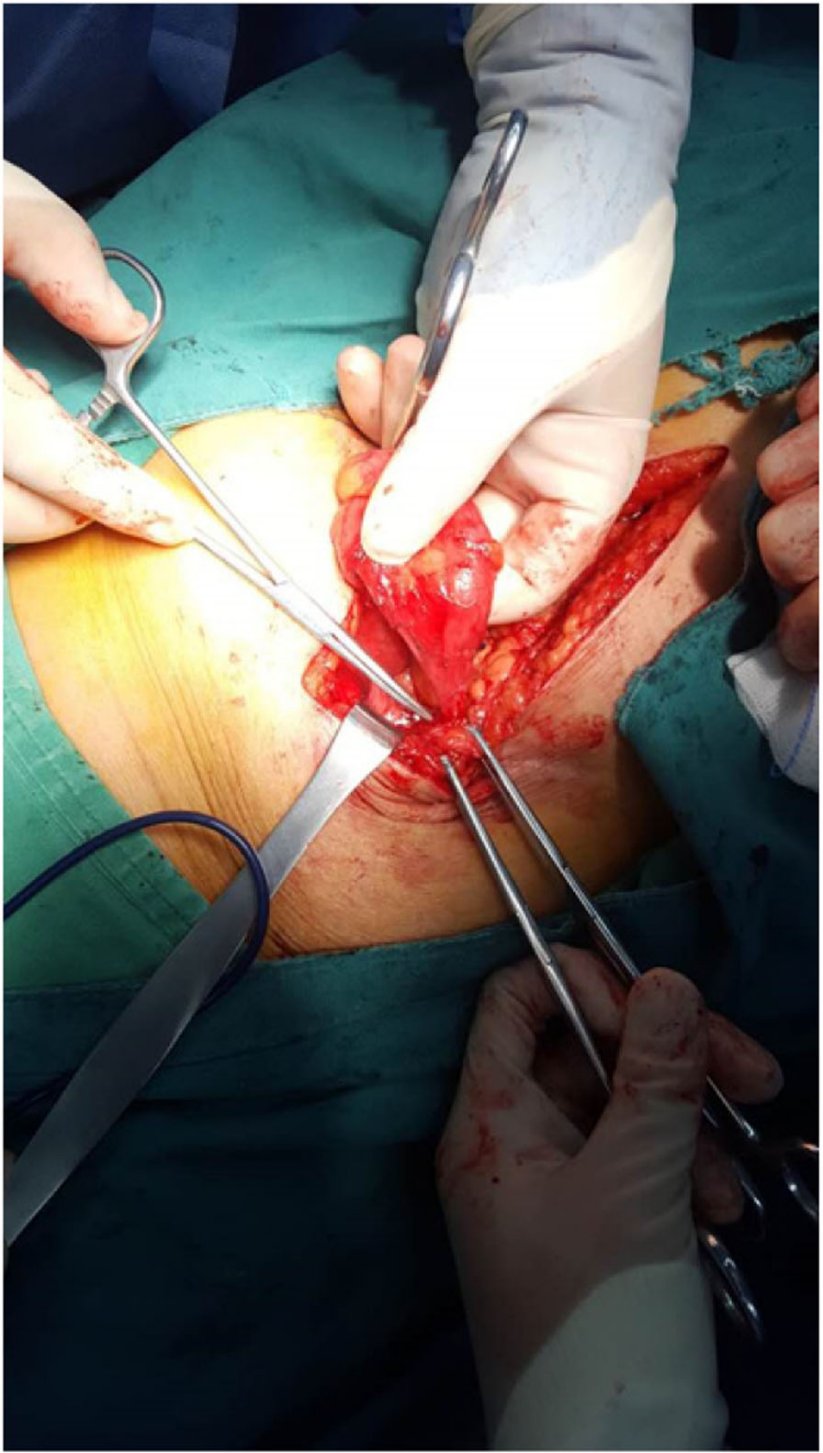
Showing loop of ileum adherent to anterior abdominal wall

**Fig. 4:**
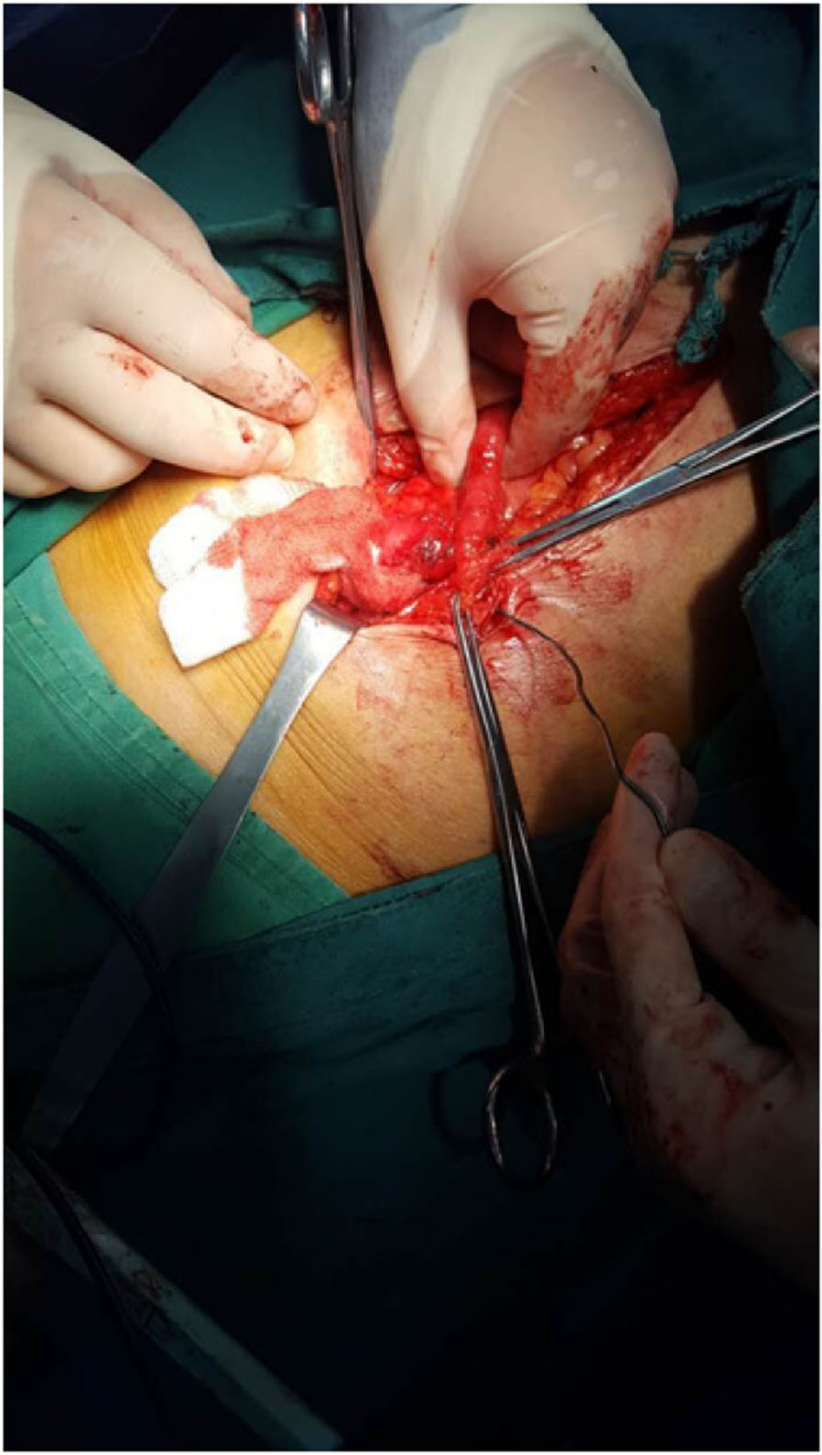
A bougie moved easily into the lumen through the cutaneous fistulas

The patient agreed to surgery after a clear preoperative conversation. She understood the operative risk factors and signed an informed consent. After bowel preparation, the patient received an exploratory laparotomy. The abdominal cavity was completely exposed, and a loop of the terminal ileum (approximately 40 cm proximal to the ileocaecal junction) was found entrapped in the internal hernia ring; this finding was consistent with the preoperative contrast image. The defect in the abdominal wall was less than 1.0 cm, and an extremely small portion of the bowel wall was stuck and could not be retrieved back into the cavity (Fig. 3). Nonetheless, this defect resulted in perforation over the loop (Fig. 4). Side-to-side ileo-ileal anastomosis was completed by utilizing a 75 mm linear stapler to remove the affected ileum segment. The internal hernia ring was closed with plication sutures instead of via mesh repair due to the patient’s small defect and infection risk. The abdominal cavity was thoroughly cleaned with saline solution, and a rubber drainage tube was placed in the pelvis. The scar tissue was removed to improve wound healing; subsequently, relaxation sutures were available to close the abdomen in layers. A final diagnosis of Richter’s hernia presenting as spontaneous ECF was reached. The patient was discharged 2 weeks after surgery without serious complications. No hernia recurrence was observed during 10 months of follow-up.

## Discussion

Richter’s hernia is named after August Gottlieb Richter; however, Fabricius Hildanus provided the earliest known record of this type of hernia in1598 [6]. Singh et al reported a rare case of spontaneous inguinal faecal fistula as a complication of incarcerated Richter’s hernia and briefly reviewed relevant literature [7].

Richter’s hernia refers to a condition in which a portion of the bowel wall is entrapped in the hernia sac without symptoms of ileus; this condition tends to occur most frequently in aged patients, particularly elderly females [1]. The most commonly reported hernia content is a loop of the distal ileum; however, any portion of the gastrointestinal tract and omentum can become incarcerated. The most common sites for Richter’s hernia are the femoral ring (36–88%), followed by the deep inguinal ring (12–36%) [8]. Richter’s hernias have been reported in other relatively rare locations, including incision and laparoscopic port insertion sites [9]. To the best of our knowledge, the presence of an ECF as a chronic complication of Richter’s hernia through a defect in the umbilical region in the absence of prior abdominal surgery is extremely rare. First, strangulation in a neglected Richter’s hernia that causes fistula is typically an urgent, rapidly developing, and critical condition with high mortality. In 1986,J.M. Horbach reviewed a series of strangulated hernias; over 30%of these hernias were Richter’s hernias, more than two-thirds of which required aggressive emergency treatment due to manifestations such as necrosis of the bowel wall [10]. This series truly reflected the rate of disease progression. Second, the majority of ECFs occurin cases involving inflammatory bowel disease or a surgical intervention history [11–14]. The literature indicates that spontaneous fistul as have been reported more frequently in developing countries, such as India and Nigeria, than in developed nations [15, 16]. Similarly, our index case involved a patient from Hong’an, a secluded mountain area in China with a primitive economy and culture. Certain factors aggravated the patient’s condition, including living alone, poverty, late presentation and a lack of appropriate therapy.

The pathophysiology of Richter’s hernia is not well understood. To date, a small abdominal wall defect with hard peripheral tissue was regarded as a key feature of the evolution and progression of this type of hernia. Increased intra-abdominal pressure due to any cause forcesa portion of the circumference of the bowel wall into the hernia sac through the internal ring. The patient in our case exhibited a susceptibility to weeping, which disturbs abdominal pressure. Spontaneous ECF is typically attributable to a blood circulation disorder with chronic bowel wall ischaemia, complicated by an abscess, that spreads through subcutaneous tissues. The external fistulous tract that is formed is ultimately caused by sustained inflammatory stimulation [17]. In 2003, Kingsnorth and Le Blane modified the types of Scarpa hernias and advisedthat cases involving Richter’s hernia could be classified into three clinical groups based on the presence or absence of intestinal obstruction [8]:(I) less than one-third of the bowel wall is involved, without ileus; (II) between one-third and two-thirds of the bowel wall is involved, with incomplete obstruction; and (III) more than two-thirds of the bowel wall is involved, with obvious obstruction symptoms. Our index case falls into group I, with no signs of acute intestinal obstruction and well-maintained luminal continuity. Preoperatively obtaining a correct diagnosis of this type is rather challenging. A significant percentage of complications resulting from incarcerated hernias are diagnosed during emergency laparotomy. In cases involving a suspected intestinal fistula, gastrointestinal imaging is useful. Such imaging can clearly indicate the locations and numbers of fistulas in the GI tract and surrounding regions, establish whether a distal intestinal obstruction exists, and provide dynamic observations of the diseased digestive tract [18]. Another type of auxiliary examination, CT, has gained abundant attention due to its advantages of being highly efficient and non-invasive [19]. During the early stages of an intestinal fistula, CT can reveal the condition of the intra-abdominal area with respect to characteristics such as effusion, abscess formation, bowel adhesion, and bowel wall oedema, among others. Moreover, imaging manifestations can exclude “frozen” abdomen and thus provide guidance to surgeons in judging surgical opportunities, choosing an appropriate incision approach, exposing the intestinal fistula clearly, and reducing operation time [20].

Richter’s hernia is a type of incarcerated hernia; similarly to other incarcerated hernias, Richter’s hernia features characteristics such as intestinal haemo dynamic disorder, ectopic bacteria, and surgical area scarring or infection. Given these characteristics, one-stage repair with mesh via exploratory laparotomy is not appropriate for the treatment of incarcerated hernia without perforation due to the risk of adhesion and ECF formation [21]. Currently, this viewpoint is facing challenges. In a retrospective descriptive study, Brandi CD et al reported that mesh repair is not correlated with the incidence of wound infection and leads to low recurrence of ECF; these findings further contribute to the controversy regarding the application of mesh repair for incarcerated hernias [22]. In our opinion, one-stage repair with mesh is adequate in the absence of peritonitis, severe ileus and/or bowel necrosis. In cases involving serious infection or combined fistula, radical debridement and suture repair without mesh are recommended. In our case, only a small piece of the anti-mesenteric bowel wall was entrapped in the hernia orifice [Fig. 3],but a fistula eventually formed, complicating the diagnostic challenge. How did this situation arise? From an anatomical standpoint, spontaneous ischaemia of the intestine isremarkable because mesenteric vessels are composed of many branches known as haemal arches that have communicating blood supplies. However, the common bowel site involved in the hernia sac is the anti-mesenteric region. In this area, vessels that provide nutrients to the intestine are derived from and perpendicular to the haemal archesbut lack a communicating blood supply and thusformarelatively ischaemic zonethat is more susceptible toenterobrosis.

## Conclusion

Richter’s hernia can be observed at any age but is particularly common among frail, elderly patients. This nonspecific clinical and laboratory findings of this condition are associated with a high misdiagnosis rate, resulting in relatively high mortality. If strangulation in Richter’s hernia is suspected, then auxiliary examinations, such as ultrasound examination, abdominal CT and gastrointestinal imaging are recommended. Timely surgical intervention is crucial for reducing mortality and improving prognosis.

## Abbreviations

CT: Computed tomography
ECF: Enterocutaneous fistula

## References

[1] Rutkow IM (2003). A selective history of hernia surgery in the late eighteenth century: the treatises of Percivall Pott, Jean Louis Petit, D August Gottlieb Richter, Don Antonio de gimbernat, and Pieter camper. Surg Clin North Am.

[2] Habib Faridi S, Siddiqui B, Amanullah Khan M, Anees A, Ali SA (2013). Suprapubic fecal fistula due to Richter’s inguinal hernia: a case report and review of literature. Iran J Med Sci.

[3] Murji A, De Gasperis-Brigante C, Leyland N (2016). Richter's hernia after laparoscopic surgery. J Minim Invasive Gynecol.

[4] Earle DB, McLellan JA (2013). Repair of umbilical and epigastric hernias. Surg Clin North Am.

[5] Cikman O, Kiraz HA, Ozkan OF, Adam G, Celik A, Karaayvaz M (2015). An extremely rare complication of Meckel's diverticulum: enterocutaneous fistulization of umbilical hernia. Arq Bras Cir Dig.

[6] Steinke W, Zellweger R (2000). Richter’s hernia and Sir Frederick Treves: an original clinical experience, review, and historical overview. Ann Surg.

[7] Ahi KS, Moudgil A, Aggarwal K, Sharma C, Singh K (2015). A rare case of spontaneous inguinal faecal fistula as a complication of incarcerated Richter's hernia with brief review of literature. BMC Surg.

[8] Kingsnorth A. The management of incisional hernia. Ann R Coll Surg Eng. 2006;88(3):252–60. doi:10.1308/003588406X106324. 10.1308/003588406X106324 PMC1963672 16719992

[9] Boughey JC, Nottingham JM, Walls AC (2003). Richter’s hernia in the laparoscopic era: four case reports and review of the literature. Surg Laparosc Endosc Percutan Tech.

[10] Horbach JM (1986). Invagination for Richter-type strangulated hernias. Trop Doct.

[11] Siegel CA, Whitman CB, Spiegel BM, Feagan B, Sands B, Loftus EV (2016). Development of an index to define overall disease severity in IBD. Gut.

[12] Scharl M, Bruckner RS, Rogler G (2016). The two sides of the coin: similarities and differences in the pathomechanisms of fistulas and stricture formations in irritable bowel disease. Gastroenterol J.

[13] Chew DKW, Choi LH, Rogers AM (2000). Enterocutaneous fistula 14 years after prosthetic mesh repair of a ventral incisional hernia: a life-long risk?. Surgery.

[14] Vrijland WW, Jeekel J, Steyerberg EW, den Hoed PT, Bonjer HJ (2000). Intraperitoneal polypropylene mesh repair of incisional hernia is not associated with enterocutaneous fistula. Br J Surg.

[15] Rattan KN, Garg P (1998). Neonatal scrotal faecal fistula. Pediatr Surg Int.

[16] Tomaszewski P (1988). Incidence of Richter’s hernia among the population of Nigeria. Wiad Lek.

[17] Elenwo SN, Igwe PO, Jamabo RS, Sonye US (2016). Spontaneous entero-labial fistula complicating Richters hernia: Report of a case. Int J Surg Case Rep.

[18] Davidson JP, Connelly TM, Libove E, Tappouni R (2016). Gastropericardial fistula: radiologic findings and literature review. J Surg Res.

[19] Bailey CR, Leeper WR, Johnson PT, Berlanstein BP, Fishman EK, Coquia SF (2016). Richter's hernia through the broad ligament: Role of computed tomography and ultrasonography in facilitating diagnosis and rapid operative repair. Surgery.

[20] Kavanagh D, Neary P, Dodd JD, Sheahan KM, O’Donoghue D, Hyland JM (2005). Diagnosis and treatment of enterovesical fistulae. Colorectal Dis.

[21] Bernard C, Polliand C, Mutelica L, Champault G (2007). Repair of giant incisional abdominal wall hernias using open intraperitoneal mesh. Hernia.

[22] Brandi CD, Roche S, Bertone S, Fratantoni ME (2016). No enterocutaneous fistula development in a cohort of 695 patients after incisional hernia repair using intraperitoneal uncoated polyproylene mesh. Hernia.

